# Therapeutic drug monitoring to personalize dosing of imatinib, sunitinib, and pazopanib: A mixed methods study on barriers and facilitators

**DOI:** 10.1002/cam4.6663

**Published:** 2023-10-30

**Authors:** Kim Westerdijk, Neeltje Steeghs, Casper S. J. Tacke, Winette T. A. van der Graaf, Nielka P. van Erp, Gerard van Oortmerssen, Rosella P. M. G. Hermens, Ingrid M. E. Desar, In collaboration with the Dutch Pharmacology Oncology Group (DPOG)

**Affiliations:** ^1^ Department of Medical Oncology Research Institute for Medical Innovation, Radboud University Medical Center Nijmegen The Netherlands; ^2^ Department of Medical Oncology Netherlands Cancer Institute, Antoni van Leeuwenhoek Amsterdam The Netherlands; ^3^ Department of Medical Oncology Erasmus MC Cancer Institute, Erasmus Medical Center Rotterdam Rotterdam The Netherlands; ^4^ Department of Pharmacy Research Institute for Medical Innovation, Radboud University Medical Center Nijmegen The Netherlands; ^5^ Patient Advocacy Group “Patiëntenplatform Sarcomen” Utrecht The Netherlands; ^6^ Department of IQ Healthcare Radboud University Medical Center Nijmegen The Netherlands

**Keywords:** barriers, facilitators, implementation, therapeutic drug monitoring, tyrosine kinase inhibitors

## Abstract

**Background:**

Personalized dosing based on measurement of individual drug levels and adjusting the dose accordingly can improve efficacy and decrease unnecessary toxicity of oncological treatment. For imatinib, sunitinib, and pazopanib, this therapeutic drug monitoring (TDM)‐guided dosing is, however, not routinely used, despite accumulating evidence favoring individualized dosing. Therefore, we aimed to identify and quantify (potential) barriers and facilitators in TDM‐guided dosing for imatinib, sunitinib, and pazopanib.

**Methods:**

We performed a mixed methods study among all stakeholders involved: patients, healthcare professionals (HCPs), pharmaceutical companies, and health insurance companies. During the first qualitative part of this study, we performed semi‐structured individual interviews and one focus group interview to identify all (potential) barriers and facilitators, and during the second quantitative part of this study, we used a web‐based survey to quantify these findings. The interviews addressed the six domains of the implementation of change model of Grol and Wensing: (1) the innovation itself; (2) the HCP; (3) the patient; (4) social context; (5) organizational context; and (6) finances, law, and governance.

**Results:**

In the qualitative study, we interviewed 20 patients, 18 HCPs and 10 representatives of pharmaceutical and health insurance companies and identified 72 barriers and 90 facilitators. In the quantitative study, the survey was responded by 66 HCPs and 58 patients. Important barriers were on the domain of the HCP, such as a lack of experience with TDM (36.4%), on the domain of the patient, such as lack of awareness of TDM (39.7%), and the processing time for measurement and interpretation of the TDM result (40.9%) (organizational domain). Important facilitators were education of HCPs (95.5%), education of patients (87.9%) and facilitating an overview of when and where TDM measurements are being performed (86.4%).

**Conclusion:**

We identified and quantified important barriers and facilitators for the implementation of TDM‐guided dosing for imatinib, sunitinib, and pazopanib. Based on our results, the implementation strategy should mainly focus on educating both HCPs and patients and on the organizational aspect of TDM.

## 1 INTRODUCTION

Tyrosine kinase inhibitors (TKIs) are oral oncologic drugs that target tyrosine kinases and have become important in the modern anticancer treatment of solid tumors.^1^, ^2^ However, their potential might benefit from dose individualization.

Currently, much attention is paid to personalized medicine in oncology, for example by investigating specific genes or mutations that can be targeted by specific drugs.^3^ However, once the appropriate drug has been selected, little attention is paid to personalize and optimize the dosage. The same holds for the treatment with TKIs, which are prescribed at fixed doses, even though large interpatient pharmacokinetic variability occurs.^4^, ^5^, ^6^, ^7^ The actual TKI exposure can differ up to 10‐fold between patients.^8^ For many TKIs, a relationship has been established between drug exposure and both efficacy and toxicity.^9^, ^10^, ^11^, ^12^, ^13^ For an increasing amount of TKIs, thresholds for drug exposure have been defined, meaning a minimum target level for optimal treatment benefit and an upper limit for preventing unnecessary toxicity.^7^, ^14^ The window between these thresholds is called the therapeutic window. It has been demonstrated that up to 52% of patients treated with TKIs have sub‐ or supratherapeutic exposure, risking decreased efficacy or unnecessary toxicity, respectively.^4^, ^5^, ^7^, ^15^

A method to optimize the dosage is trough routinely measured drug concentrations in plasma and adjusting the dose accordingly, which is called therapeutic drug monitoring (TDM).^16^ A plasma trough concentration (C_trough_) outside the prespecified therapeutic window will lead to dose adjustments with the aim to give each individual patient the most optimal exposure level. TDM‐guided dosing is routinely used to optimize the treatment of patients treated with, for example antibiotics, immunosuppressants, or anti‐epileptic drugs.^17^, ^18^ Furthermore, it is commonly used within oncology for treatment with methotrexate, busulfan, and mitotane.^19^, ^20^, ^21^ However, for TKIs, TDM‐guided dosing is less common, despite accumulating evidence. Most evidence of the benefits for TDM‐guided dosing is available for imatinib, sunitinib, and pazopanib specifically. Furthermore, the feasibility to reach drug exposure within the therapeutic window with TDM‐guided dosing has been demonstrated for these drugs.^4^, ^10^, ^22^, ^23^, ^24^ In a recent study, the percentage of patients with a drug exposure below target decreased from 70.4% to 39.6%, 44.3% to 14.3%, and 26.7% to 16.7% for imatinib, sunitinib, and pazopanib, respectively, using TDM‐guided dosing.^25^ Therefore, TDM‐guided dosing is considered appropriate and implementable for these three TKIs. Still, its routine use in clinical practice is limited.^7^, ^26^, ^27^

To gain insight why innovations that lead to better treatment outcomes are not implemented into clinical practice instantly, various theories and models are available, for example the implementation of change model by Grol and Wensing.^28^ The first steps in this model are to explore and quantify existing and potential barriers and facilitators in the implementation of an innovation, since these barriers may require different strategies in order to overcome them. In the implementation of innovations, multiple stakeholders are involved, and therefore, it is important to identify barriers and facilitators for all stakeholders.

Currently, studies on barriers and facilitators of TDM‐guided dosing for TKIs are lacking. Identification and quantification of these barriers and facilitators can be used to develop a tailored strategy for implementation of TDM‐guided dosing.^28^ Therefore, we performed a mixed methods study among all stakeholders to identify and quantify existing and potential barriers and facilitators in the current use of TDM‐guided dosing for patients with solid tumors treated with imatinib, sunitinib, and pazopanib, which may also serve as a model for other oral oncolytics.

## 2 METHODS

### 2.1 Study design

We used a mixed methods design. In order to identify (potential) barriers and facilitators in the implementation of TDM‐guided dosing for TKIs, we performed a qualitative study using semi‐structured individual interviews among all stakeholders involved and one focus group interview among patients. Next, we performed a quantitative study using a web‐based survey to quantify the findings from the interviews and to assess the importance of the identified barriers and facilitators. This study was performed in line with the Dutch Code of Conduct for Research Integrity and the Consolidated criteria for Reporting Qualitative studies (COREQ).^29^ The study protocols for both the interview study and survey were approved by the local Medical Ethics Committee of the Radboudumc in Nijmegen, the Netherlands (dossier number 2019–5631 and 2022–16,004, respectively).

### 2.2 Study population

#### 2.2.1 Qualitative interviews

Four groups of stakeholders were identified in the implementation of TDM‐guided dosing: patients, healthcare professionals (HCPs; both medical oncologists and hospital pharmacists), pharmaceutical companies, and health insurance companies. Among them, we performed semi‐structured individual interviews and one focus group interview with patients.

Patients were included if they had solid tumors and were treated with imatinib, sunitinib, or pazopanib. Exclusion criterium was a lack of understanding of the Dutch language. Experience with TDM was not mandatory. Patients were invited for the interview via an email sent by the Dutch Patient Advocacy Groups.

HCPs with experience in treatment of patients with solid tumors with imatinib, sunitinib, or pazopanib were invited by email to participate in the interview. Experience with TDM was not mandatory. We invited HCPs from different types of hospitals (academic, teaching, and non‐teaching hospitals; *n* = 11) distributed over four regions in the Netherlands (north, east, south, and west).

The two pharmaceutical companies that produce imatinib, sunitinib, and pazopanib are Novartis and Pfizer. Two representatives of each company were invited by email for an interview. Two representatives of four large health insurance companies in the Netherlands (CZ, VGZ, Menzis, and Achmea) were invited by email to participate in the interview as well.

#### 2.2.2 Quantitative research: Survey

To quantify the barriers and facilitators found, an electronic survey was performed among HCPs and patients in the Netherlands.

All Dutch medical oncologists and hospital pharmacists received a hyperlink to the questionnaire through the e‐mail service of the Dutch Society of Medical Oncology and Dutch Association of Hospital Pharmacists, respectively. The introduction of the questionnaire requested all medical oncologists who treat patients with imatinib, sunitinib, and pazopanib and all hospital pharmacists who are known with TDM‐guided dosing to fill in the questionnaire. A reminder to the questionnaire was sent four times. Completion of the questionnaire took approximately 20 min.

The survey among patients was performed only in patients with gastrointestinal stromal tumor (GIST) treated with imatinib, since these patients are more familiar with prolonged TKI treatment and more experienced with TDM. Patients with GIST who received active treatment with imatinib and had experience with TDM‐guided dosing were invited to fill in the questionnaire via an email with a hyperlink to the questionnaire sent by the Dutch Patient Advocacy Group. A patient information sheet was attached to the email, explaining the goal of the study. Patients could open the questionnaire by clicking on the hyperlink attached to the email. A reminder to the questionnaire was sent once, and completion took approximately 15 min.

### 2.3 Data collection

#### 2.3.1 Qualitative interviews

All interviews were performed with an interview guide based on the theoretical model of Grol and Wensing and addressed the six domains of this model: (1) the innovation itself; (2) the HCP; (3) the patient; (4) the social context; (5) the organizational context; and (6) finances, law, and governance.^30^, ^31^ According to the COREQ criteria for qualitative research, interviews with patients and HCPs were performed until data saturation was reached, meaning that additional interviews do not lead to new information.^29^ This means that the number of interviews is open at the beginning of the study and is determined by the data collected. Due to the small number of health insurance companies and pharmaceutical companies involved in this project, representatives of all companies involved were interviewed.

All participants who responded to the invitation were contacted by telephone or email by one of the researchers for additional information. All participants received written information explaining the goal of the study and the process of the interview. All participants signed informed consent and gave permission for audiotaping the interviews. None of the researchers were involved in the treatment of the patients.

The interviews were structured as follows: First, participants were asked to describe their experience with TDM‐guided dosing. Then, open‐ending questions addressing one of the domains were asked. We explored barriers and facilitators in detail when they came up during the interview. If no new input came up, a new domain was introduced. The individual interviews were conducted by a trained researcher (KW). The focus group interview with patients was conducted by two trained researchers (KW and RH).

For the patients, before the interviews were conducted, basic patient characteristics were collected by means of a short questionnaire. This questionnaire included questions about age, sex, home setting, occupational status, treatment setting, and type of TKI treated with.

#### 2.3.2 Survey

The barriers and facilitators identified in the interviews were converted into a web‐based questionnaire for HCPs and patients using Limesurvey (https://manual.limesurvey.org).

For HCPs, the first part of the questionnaire consisted of nine questions about the HCPs personal characteristics (e.g., age and years of experience) and clinical setting. The second part of the questionnaire addressed the barriers and facilitators identified in the interviews and consisted of 81 theses, scored on a five‐point Likert scale (“strongly agree,” “agree,” “neutral,” “disagree,” and “strongly disagree”). The majority of questions were to be answered by both medical oncologists and hospital pharmacists and some questions were for medical oncologists or hospital pharmacists only.

For patients, electronic informed consent was necessary to start the actual questionnaire. After informed consent was provided, the first part of the questionnaire contained 13 questions about the patients' characteristics (e.g., age, gender, and education) and treatment with imatinib. The second part of the questionnaire consisted of 43 theses and scored similar to the questionnaire of HCPs. These were the barriers and facilitators that were either mentioned by patients during the interviews or were on the patient domain. Since this was not a validated questionnaire, it was pilot tested by six patients for clarity, understanding, and language, after which some minor changes were made.

At the end of the questionnaire, all survey participants could mention additional barriers and facilitators and state comments.

### 2.4 Analysis

#### 2.4.1 Qualitative interviews

All interviews were audiotaped and transcribed literally in Microsoft Word afterwards. The transcripts were imported in Atlas.Ti (version 8.4.20 Atlas.Ti Scientific Software Development GmbH; Berlin, Germany) and analyzed using framework content analysis.^32^ The framework was based on the six domains of Grol and Wensing.^31^ First, two trained researchers (KW and CT) independently coded all barriers and facilitators mentioned in the interviews. Discrepancies were discussed until consensus was reached. If no consensus was reached, a third researcher (RH) was consulted. Second, comparable descriptive codes were combined and redefined into specific subthemes. The subthemes were merged into the six broader domains of the model of Grol and Wensing using axial coding.

#### 2.4.2 Survey

The questionnaire data were gathered in Limesurvey and exported into IBM SPSS statistics for Windows, version 27.0 (IBM Corp., Armonk, NY, USA). We included questionnaires of which at least 50% of the questions were completed. We used descriptive statistics to describe characteristics of the HCPs and patients. For the 5‐point Likert scale theses, we calculated the percentage of HCPs and patients that agreed with the theses by combining the answers “strongly agree” and “agree.” We considered a barrier or facilitator to be important when more than one‐third (33%) of the respondents (strongly) agreed. In case the survey yielded a high number of important barriers and facilitators, the 10 most important barriers and facilitators for both HCPs and patients were selected.

## 3 RESULTS

### 3.1 Study population

Between September 2019 and February 2021, we performed 1 focus group interview (*n* = 12) and 8 individual interviews with patients, 18 individual interviews with HCPs (*n* = 9 medical oncologists, *n* = 9 hospital pharmacists), 2 interviews with 2 representatives from the pharmaceutical companies during each interview, and 4 interviews with a total of 6 representatives of Dutch health insurance companies. Data saturation was reached after interviewing 18/20 patients and 16/18 HCPs, respectively. Basic patient characteristics are summarized in Table 1. TDM‐guided dosing was applied in 50% of the patients in the interviews. HCPs from 10 different hospitals participated in the interviews (see Table S1).

**TABLE 1:** Characteristics of interview patients^a^.

Patients (*n*)	20
Age, median (range) in years	64 (29–85)
Time since diagnosis, median (range) in months	104 (12–230)
Duration of treatment with TKI in months, median (range)	36 (2–213)
Gender, *n* (%)	
Male	14 (70)
Female	6 (30)
Treatment, *n* (%)	
Imatinib	11 (55)
Sunitinib	4 (20)
Pazopanib	5 (25)
Use of TDM, *n* (%)	
Yes	10 (50)
No	6 (30)
Unknown	4 (20)
Treatment setting, *n* (%)	
Adjuvant	5 (25)
Metastatic	15 (75)
Nationality, *n* (%)	
Dutch	19 (95)
Chinese	1 (5)
Educational level^b^, *n* (%)	
Low	7 (35)
Intermediate	5 (25)
High	6 (20)
Unknown	2 (10)
Occupational status, *n* (%)	
Unemployed	2 (10)
Employed	4 (20)
Incapacitated	6 (30)
Retired	6 (30)
Unknown	2 (10)
Household, *n* (%)	
Partner	12 (60)
Partner and children	5 (25)
With parents	1 (5)
Unknown	2 (10)

Abbreviations: TDM, therapeutic drug monitoring; TKI, tyrosine kinase inhibitor.

^a^ Two patients failed to return the questionnaire; therefore, some information is missing.

^b^ Low educational level: primary education, lower general secondary education, preparatory secondary vocational education; intermediate educational level: secondary vocational education, higher general secondary education, pre‐university education; high educational level: higher vocational education, academic education.

The HCP survey yielded a total of 74 responses, of which 66 (89%) were completed for at least 50% and were included in the analysis. Among the responders were 33 medical oncologists and 33 hospital pharmacists from 45 hospitals (out of a total of 57 hospitals, 79%). Characteristics and clinical setting of the HCPs are shown in Table 2.

**TABLE 2:** Baseline characteristics healthcare professionals survey.

	Medical oncologists (*n* = 33)	Hospital pharmacists (*n* = 33)	Total (*n* = 66)
Gender, *n* (%)			
Male	15 (45.5)	19 (57.6)	34 (51.5)
Female	18 (54.5)	14 (42.4)	32 (48.5)
Age, median (range)	46 (35–61)	38 (30–57)	41 (30–61)
Years of experience, median (range)	13 (0–32)	7 (1–25)	10 (0–32)
Type of hospital working in, *n* (%)			
Academic	16 (48.5)	6 (18.2)	22 (33.3)
Teaching	14 (42.4)	18 (54.5)	32 (48.5)
Non‐Teaching	3 (9.1)	9 (27.3)	12 (18.2)
Experience with TDM, *n* (%)			
Yes	27 (81.8)	28 (84.8)	55 (83.3)
No	6 (18.2)	5 (15.2)	11 (16.7)
Number of patients or TDM requests annually, median (range)	10 (0–200)	20 (1–4473)	‐

Abbreviation: TDM, therapeutic drug monitoring.

The patient survey yielded 67 responses, of which 58 (87%) were completed for at least 50% and included in the analyses. Characteristics of the patients are shown in Table 3.

**TABLE 3:** Baseline characteristics patients survey.

Patients (*n*)	58
Age, median (range) in years	68 (34–85)
Gender, *n* (%)	
Male	24 (41.4)
Female	34 (58.6)
Country of birth, *n* (%)	
Netherlands	54 (93.1)
Indonesia	2 (3.4)
Australia	1 (1.7)
Unknown	1 (1.7)
Educational level^a^, *n* (%)	
Low	12 (20.7)
Intermediate	14 (24.1)
High	32 (55.2)
Occupational status, *n* (%)	
Unemployed	2 (3.4)
Employed	14 (24.1)
Incapacitated	7 (12.1)
Retired	35 (60.3)
Household, *n* (%)	
Partner	41 (70.7)
Partner and children	6 (10.3)
Children	1 (1.7)
One‐person	10 (17.2)
Years since diagnosis, median (range)	6 (0–21)
Duration of treatment with TKI in months, median (range)	29 (2–260)
Treatment setting, *n* (%)	
Neo‐adjuvant	2 (3.4)
Adjuvant	19 (32.8)
Metastatic	36 (62.1)
Unknown	1 (1.7)
Dose adjustment during treatment with imatinib, *n* (%)	
Yes	29 (50.0)
No	29 (50.0)

Abbreviations: TKI, tyrosine kinase inhibitor.

^a^ Low educational level: primary education, lower general secondary education, preparatory secondary vocational education; intermediate educational level: secondary vocational education, higher general secondary education, pre‐university education; high educational level: higher vocational education, academic education.

### 3.2 Barriers and facilitators

In the interviews, we identified a total of 72 barriers and 90 facilitators in all 6 domains of the framework. Within each domain, we grouped the barriers and facilitators into themes. Most barriers were present in the domain of the HCP (*n* = 23), the patient (*n* = 17), and organizational context (*n* = 13). Most facilitators were present in the domain of organizational context (*n* = 21), the innovation itself (*n* = 19), and the patient (*n* = 18). An overview of all barriers and facilitators is shown in Tables 4 and 5, respectively. Illustrative quotations from the interviews are shown in Figure S1.

**TABLE 4:** Barriers interviews.

Innovation itself (*n* = 2)	Healthcare professionals (*n* = 23)	Patients (*n* = 17)	Social context (*n* = 6)	Organizational context (*n* = 13)	Finances, law and governance (*n* = 11)
Implementation in guidelines TDM has not been implemented in clinical practice guidelines/local protocols. Lack of practical guidelines on use of TDM.	Awareness HCP is unaware of TDM. Hospital pharmacist is unaware of availability of online TDM monographs. Knowledge Lack of knowledge of TDM. Inability to find proper background sources. Interpretation of C_trough_ can be difficult. Laboratory worker does not know what to do with blood sample and where to send it to. Attitude Insufficient supporting evidence. HCP questions added value of TDM. HCP does not see added value of TDM being performed on a routinely basis. Reluctance to perform dose reduction in cases of high C_trough_ and limited toxicity. HCP considers TDM to be expensive. Assumption that health insurance company does not pay for other than standard TKI dosages. Assumption that treatment will not be paid for by health insurance company in off‐label use of TKI. Assumption that health insurance company will not facilitate implementation of TDM due to costs. Resistance of HCP to deviate from clinical practice guidelines in which TDM is absent. Behavior HCP being stuck in routine and TDM has not been incorporated in that routine yet. Communication Pharmacy emphasized costs of TKI to patient due to dose increase based on TDM. Experience Lack of experience and expertise with TDM. Hospital pharmacist experiences difficulties in formulating dose advice based on C_trough_. Translating C_trough_ to clinical dose advice might be difficult. Workload High workload. Inability to stay informed of latest innovations due to rapidly emerging scientific evidence. Time consuming for hospital pharmacist to retrieve latest intake of TKI.	Awareness Patient is unaware of TDM. Creating awareness of TDM through patient advocacy groups can cause anxiety. Patient is unaware of possibility to perform TDM blood sampling close to home. Knowledge TDM remains abstract for patients. Attitude Patient questions added value of TDM (especially in case of high C_trough_). Patient is under the impression that implementation of TDM is driven by financial stimuli. Fears Stress for C_trough_ result. Patient fears that dose escalation results in an increase in adverse events. Patient fears that dose escalation results in limitations in daily life. Fear that dose reduction in case of high C_trough_ results in loss of efficacy. Fear of lack of efficacy in case of low C_trough._ Quality of life Experienced decrease in quality of life due to increase in toxicity. Technical aspects Dose adjustments complicated for patients. Dose escalation increases the number of pills that patients have to take. Reason why C_trough_ measurement requires change of moment of TKI intake might be confusing. Patient forget to skip TKI intake on day of C_trough_ measurement. Result of C_trough_ is not available yet during appointment with medical oncologist.	Collaboration In smaller hospitals where no hospital pharmacist is available, it is more difficult to consult an external hospital pharmacist. Collaboration is insufficient in some regions/hospitals. Local hospital pharmacist was bypassed by treating physician in performing TDM and therefore was not able to provide dose advice. HCP (colleagues) It is hard to initiate implementation of TDM when this is not routine practice in colleagues. Increase in costs due to TDM must be explained to the board (especially in smaller hospitals). Relatives Fear of dose adjustments (either increase in adverse events due to dose escalation or lack of efficacy in case of dose reduction).	Nationwide organization Extra workload of external C_trough_ measurements. Absence of clear contact person in case of questions regarding TDM. Order for C_trough_ measurement Order for C_trough_ measurement is not available in electronic patient file. Order for C_trough_ measurement was absent at the moment of blood sampling. Each hospital uses a different laboratory form. Blood sampling Last TKI intake was not registered when blood sample was taken. Timing of C_trough_ measurement can be challenging since some patients take TKI in fasted state. Patient is not instructed to skip TKI prior to C_trough_ measurement. Pharmacy Processing time of C_trough_ results takes too long. Information regarding last TKI intake could be lost when sending blood sample to external hospital pharmacy. Hospital pharmacist does not have all clinically relevant information necessary for dose advice. Availability of only one dedicated hospital pharmacist. Pharmacy gave wrong intake instructions to patient when handing over TKI.	Possible increase in costs Dose escalation can result in an increase in costs (due to increased costs of medication). Implementation of TDM is hindered by expectations regarding costs. Costs of C_trough_ measurement are especially at the expense of pharmacy. External C_trough_ measurement is more expensive compared to C_trough_ measurement in own hospital. In smaller hospitals less resources are available. Financial compensation Financial compensation for TDM is unclear. Expenses of hospital will increase due to blood samples while reduction in costs resulting from TDM will only benefit health insurance companies. Pharmaceutical company Pharmaceutical company will not invest in drug with expiring patent. Pharmaceutical company does not warrant correctness of leaflet of medication in case of off‐label use. Fear of pharmaceutical company that TDM complicates treatment for medical oncologists. Limited international use of TDM results in lack of enthusiasm with board of pharmaceutical company, making it more difficult for national pharmaceutical company to facilitate implementation of TDM.

Abbreviations: C_trough_, trough concentration; HCP, healthcare professional; TDM, therapeutic drug monitoring; TKI, tyrosine kinase inhibitor.

**TABLE 5:** Facilitators interviews.

Innovation itself (*n* = 19)	Healthcare professionals (*n* = 11)	Patients (*n* = 18)	Social context (*n* = 6)	Organizational context (*n* = 21)	Finances, law and governance (*n* = 15)
Evidence Availability of evidence of exposure‐response and exposure–toxicity relationship. HCP requires prospective studies confirming positive effect on treatment outcome. (Evidence that) TDM is cost‐effective. Implementation in guidelines Implementing TDM in clinical practice guidelines/local protocols. To apply a uniform methodology for use of TDM in all hospitals. Development of practical guidelines on use of TDM. Advantages of dose adjustments Optimizing treatment with TKI (improving efficacy and preventing/decreasing toxicity). Individualizing treatment of patient. TDM enables dose optimization in an early stage. Being able to start with a low dose and increase the dose carefully based on C_trough_ in case of fragile patients. Dose reduction decreases the number of pills patients have to take. Advantages monitoring To objectify whether current TKI dose results in adequate exposure. Evaluation of therapy adherence. Monitoring TKI exposure in case of interacting co‐medication. Hospital pharmacist also evaluates co‐medication when performing TDM. Tools TDM monographs are available. Availability of MW‐Pharm models would be of added value. Dried blood spot would increase accessibility and use of TDM. Other TDM is easy to perform.	Awareness Increasing awareness of TDM (especially among medical oncologists. Informing HCP properly about the processing time of the C_trough_ results. Knowledge Availability of background literature on TDM. Implementing TDM in educational programs of HCPs (preferably by peers). Attitude Confidence that C_trough_ result is representative reflection of treatment. Expectation that HCP is open‐minded about TDM. Persuasion of the added value of TDM. Communication Sufficient explanation of possible advantages and disadvantages of dose escalation. HCP informs patients of TDM result. Experience Interpretation of C_trough_ becomes easier when gaining experience. Other HCP receives practical advice with C_trough_ result.	Awareness Increasing awareness of TDM through various media. Embursing patient empowerment, for example, via patient advocacy groups. Attitude Patient is convinced of added value of routine TDM. Patient considers possible reduction in costs to be important. Confidence that C_trough_ result is representative reflection of treatment. Patient–HCP relationship Confidence in HCP. Communication Instructing patients thoroughly on when to take TKI prior to TDM measurement. Reminding patients to skip TKI prior to C_trough_ measurement by hospital or patients themselves (e.g., via notes). Use of simple and clear explanation when educating patients on TDM (e.g., drawings or pictures). Discussing expectations of patients of TDM (for example processing time of C_trough_ result, possible adverse events). Quality of life TDM increases quality of life. TDM increases patient satisfaction. Motivations/needs TDM increases experience of receiving maximum treatment effort. TDM confirms adequate treatment dose. TDM increases patients’ compliance to treatment. TDM increases patient involvement. C_trough_ helps to reassure that dose reduction is safe regarding efficacy in case of toxicity and high C_trough._ Patient experiences positive effect of TDM (for example to take TKI with food instead of in fasted state).	Collaboration Good collaboration between HCPs within a hospital. Good collaboration between different hospitals. Relatives Partner/family is convinced of added value of TDM. HCP Help of colleagues in case of questions about TDM. Belief in added value of TDM by entire medical profession. Use of TDM in neighboring hospitals can stimulate applying TDM in own hospital (peer pressure).	Nationwide organization To provide an overview of where C_trough_ measurement is being performed. Use of already existing logistic routes to hospitals where C_trough_ is being measured (if possible). Centralizing measurement of C_trough._ Availability of clear guidelines of timing TKI intake and C_trough_ measurement. Generic instruction to patients about moment of TKI intake so patients do not have to skip TKI intake prior to C_trough_ measurement. Availability of clear contact person in case of questions regarding TDM. Local organization Possibility of blood sampling close to patients’ home. Possibility to obtain TKI at local pharmacy or to deliver at patients’ home in case of dose adjustment due to TDM. Presence of a hospital pharmacist dedicated to TDM. Assigning clear contact person to each patient in case of questions concerning TDM. Order for C_trough_ measurement Ordering and reporting results of C_trough_ measurement should be simplified (e.g., the availability of an electronic order). Addition of dose advice formulated by hospital pharmacist to C_trough_ result in electronic patient file. Blood sampling Possibility of collecting blood samples a few days prior to the analysis of samples in hospital pharmacy. Possibility of collecting blood samples well ahead of appointment with medical oncologist. Reporting latest TKI intake thoroughly when blood sample is taken (e.g., by educating all involved). Collecting all blood samples by one dedicated person. Taking blood sample exactly 24 h after last TKI intake (to decrease necessary effort for hospital pharmacist). Pharmacy Quicker availability of result of C_trough_ measurement (e.g., by more frequent analysis or by developing assay in local hospital). Possibility to measure exposure of multiple TKIs at once. Other Informing patient about C_trough_ measurement by pharmacy when handing over TKI for the first time. Availability of pills with different dosages of TKIs.	Possible reduction in costs Decreasing costs due to dose reduction based on C_trough_ measurement (due to either dose reduction itself or decrease in adverse events). Increasing TKI exposure with alternative methods (e.g., CYP3A4 boosting or ingestion of TKI with food). Increasing effectiveness of TKI by optimizing exposure can delay possibly more expensive subsequent line of treatment. Reducing travel expenses by enabling local blood sampling. Relative costs of TDM Costs of TDM are low, especially relative to entire oncologic treatment with TKIs. Total costs of TDM for hospital are low due to limited number of patients. Financing of TDM‐guided dosing should be arranged by hospital as a whole instead of by each individual department. Convincing HCPs of added value of TDM reduces concerns about costs. Financial compensation Arranging compensation for TDM costs, for example, via health insurance companies or via the Dutch Health Authority. Obtaining compensation for costs of TDM can be more easy when implementing TDM in clinical practice guidelines. Health insurance company can incorporate TDM‐guided dosing as a quality requirement. Pharmaceutical company TDM can be financially interesting for pharmaceutical company considering increased effectiveness and extended treatment with TKI. Paying costs of TDM (partially) by pharmaceutical company. Facilitating implementation of TDM by pharmaceutical company by adding TDM to SMPC. Other Dose increase is less expensive when patent is expired.

Abbreviations: C_trough_, trough concentration; CYP3A4, Cytochrome P450 3A4; HCP, healthcare professional; TDM, therapeutic drug monitoring; TKI, tyrosine kinase inhibitor.

**FIGURE 1:**
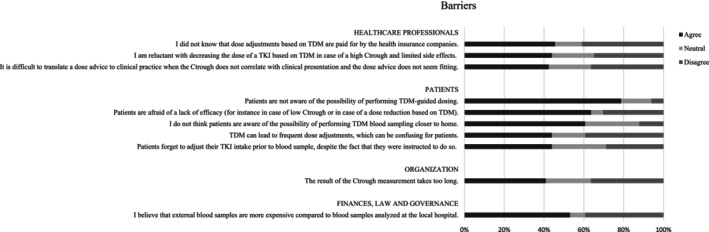
Ten barriers with highest percentage of agreement for HCPs. C_trough_, trough concentration; HCP, healthcare professional; TDM, therapeutic drug monitoring; TKI, tyrosine kinase inhibitor.

**FIGURE 2:**
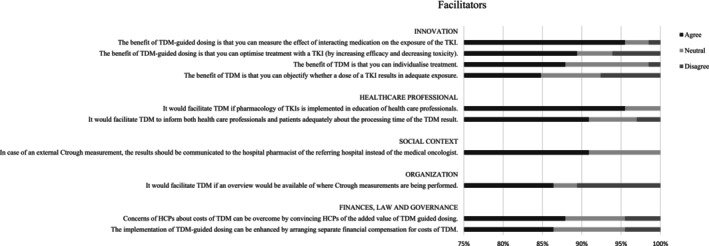
Ten facilitators with highest percentage of agreement for HCPs. C_trough_, trough concentration; HCP, healthcare professional; TDM, therapeutic drug monitoring; TKI, tyrosine kinase inhibitor.

**FIGURE 3:**
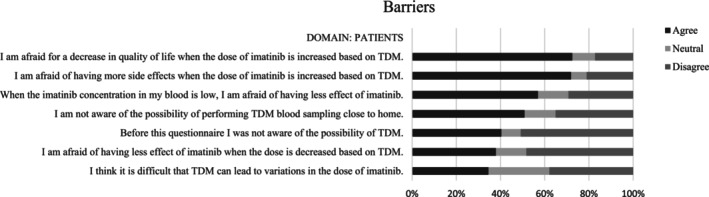
Important barriers for patients. TDM, therapeutic drug monitoring.

**FIGURE 4:**
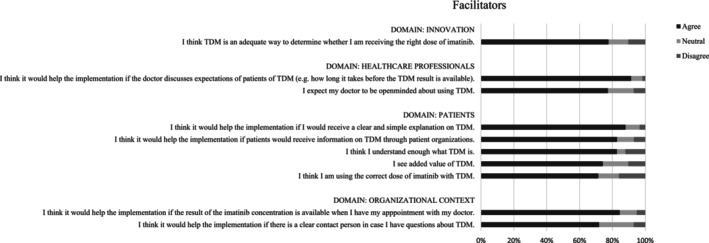
Ten facilitators with highest percentage of agreement for patients. TDM, therapeutic drug monitoring.

The responses to the theses in the questionnaire for HCPs and patients are shown in Tables 2 and 3, respectively. The 10 barriers and 10 facilitators that yielded the highest percentage of agreement in HCPs are shown in Figure 1 and Figure 2, respectively. The important barriers and 10 facilitators with highest percentage of agreement in patients are shown in Figures 3 and 4, respectively. Results from both the interviews and the survey will be discussed further by domain.

#### 3.2.1 Domain: Innovation itself (TDM)

The majority of HCPs in the survey recognized benefits of TDM‐guided dosing, such as the opportunity to individualize treatment (87.9%). Especially in fragile patients, TDM‐guided dosing enables a physician to start at a low dose and increase the dose carefully based on C_trough_ levels (68.2%). Patients in the survey also responded that TDM can help determine whether they are treated with the right dose (77.6%) and that the effect of treatment increases (69.0%) and the side effects decrease (60.3%) with TDM. Health insurance companies and pharmaceutical companies acknowledged these benefits of TDM in the interviews as well.

Many HCPs (40.9%) stated that prospective studies proving the added value of TDM‐guided dosing on treatment outcome, such as improved survival, are required before they would perform TDM‐guided dosing in routine clinical practice. The HCPs in the interviews mentioned that they did believe that TDM‐guided dosing could be of added value in specific cases, but that they were not convinced of the added value of performing TDM‐guided dosing in all patients in routine care. HCPs, patients and the pharmaceutical companies, mentioned during the interviews that TDM‐guided dosing has not been implemented in clinical guidelines and/or local protocols and that this could cause both lack of awareness of TDM‐guided dosing, but also decrease the willingness of HCPs to perform TDM‐guided dosing. However, this was not confirmed by the survey. All health insurance companies in the interviews believed it could facilitate TDM to prove that TDM is cost‐effective.

#### 3.2.2 Domain: Healthcare professional

HCPs agreed that they lack experience with TDM‐guided dosing with TKIs (36.4%). Furthermore, many medical oncologists are reluctant to decrease the dose in case of a high C_trough_ and limited side effects (43.9%) and agreed that it can be difficult to translate a dose advice to clinical practice when the C_trough_ does not correlate with clinical presentation and the dose advice does not seem appropriate (42.4%). In the interviews, some medical oncologists mentioned patients that had severe side effects but low C_trough_ levels. As a result, the hospital pharmacist advised to increase the dose, though this was not feasible in clinical practice. Many HCPs (45.5%) seem to be unaware that TDM‐guided dose adjustments within label are covered by health insurance companies. The patient survey revealed no relevant barriers on the HCP domain.

The HCP survey results also showed that implementation of TDM would be facilitated if pharmacology of TKIs is implemented in education of HCPs (95.5%). Both HCPs (90.9%) and patients (91.4%) in the survey stated that they should be adequately informed about the processing time of the TDM result.

#### 3.2.3 Domain: Patient

Both HCPs and patients in the survey responded that patients are not aware of the possibility of performing TDM‐guided dosing (78.8% and 39.7%, respectively) or of the possibility of performing blood sampling in a local hospital closer to home (60.6% and 50.0%, respectively). In order to reliably calculate a C_trough_ level, it is important that the blood sample is taken after the time it takes to reach the maximum drug concentration (T_max_). HCPs in the survey stated that patients often forget to adjust timing of TKI intake to ensure the blood sample is taken after T_max_ (43.9%). This was not found in the patient survey (25.9%), however. HCPs reported that patients fear lack of efficacy of treatment (in case of a low C_trough_ or in case of a dose reduction based on TDM) (63.6%) and that repetitive dose reductions due to TDM can be confusing for patients (43.9%). Patients in the survey also agreed that they fear lack of efficacy in case of a low C_trough_ (56.9%) or a dose reduction (37.9%) and that dose reductions can be difficult (34.5%). During the interviews, patients mentioned that performing TDM causes awareness of low C_trough_ levels. This is a positive thing whenever a dose increase is feasible. However, when a dose increase is not feasible, for example due to previous toxicity at a higher dose, patients are made aware of low C_trough_ levels and potential suboptimal treatment. This can cause anxiety and stress.

According to both HCPs and patients, TDM‐guided dosing can increase patients' involvement in‐ (60.6% and 63.8%, respectively) and satisfaction about their treatment (51.5% and 56.9%, respectively). The value of patient advocacy groups in creating awareness of TDM among patients is recognized by both HCPs (54.5%) and patients (82.8%).

#### 3.2.4 Domain: Social context

Implementation of TDM in a certain hospital can be enhanced by the implementation of TDM‐guided dosing in surrounding hospitals (63.6%). In case of an external C_trough_ measurement, interpretation of external TDM result and subsequent dose advice is the responsibility of the local hospital pharmacist and they should not be bypassed by the medical oncologist (90.9%).

None of the barriers identified in the interviews on the domain of social context were considered to be important by the survey respondents.

#### 3.2.5 Domain: Organizational context

Although many barriers were mentioned on the domain of the organizational context in the interviews (Table 3), this was not confirmed in the survey. HCPs in the survey did only agree that the result of the C_trough_ measurement takes too long (40.9%). Currently, the processing time for the measurement and interpretation of the C_trough_ result is about 1–2 weeks.

It would facilitate TDM if an overview would be available of when and where C_trough_ measurements are being performed (86.4%). During the interviews, HCPs mentioned that this would allow them to adjust timing of blood samples in order to obtain results more quickly. Patients in the survey stated that they would prefer blood sampling well before the appointment with the medical oncologist, to ensure that the TDM result is available (84.5%).

#### 3.2.6 Domain: Finances, law, and governance

The actual costs of an individual TDM measurement are between 60 and 75 euros. In the survey, 63.6% of HCPs answered this question correctly, though 24.3% thought that costs were higher. HCPs in the survey assumed that external blood samples are more expensive compared to blood samples analyzed at the treating hospital (53.0%).

The HCP survey revealed that the implementation of TDM‐guided dosing can be enhanced by arranging separate financial compensation for costs of TDM measurements (86.4%), by incorporating TDM in the drug label by the pharmaceutical companies (83.3%) or by incorporating it as a quality requirement by health insurance companies (83.3%). However, 87.9% of HCPs agreed that concerns of HCPs about costs could be overcome by convincing them of the added value of TDM. Both the health insurance and pharmaceutical companies in the interviews believed that arranging financial compensation for TDM measurements would facilitate its implementation and that this would be more straightforward when TDM is implemented into clinical guidelines or in the drug label.

No barriers or facilitators for law and governance were mentioned during the interviews.

## 4 DISCUSSION

In this mixed methods study, we identified many important barriers and facilitators in the implementation of TDM‐guided dosing for patients with solid tumors treated with imatinib, sunitinib, and pazopanib in routine clinical practice. Barriers and facilitators were present in all domains of the framework of Grol and Wensing and among all stakeholders.^30^, ^31^ Important barriers were a lack of experience of HCPs with TDM, difficulties in translating a dose advice to clinical practice, a lack of awareness of TDM among patients and the processing time for the measurement and interpretation of the C_trough_ result. Important facilitators were the implementation of pharmacology of TKIs in the education of HCPs, education of patients and to provide an overview of when and where C_trough_ measurements are being performed. Knowledge of these barriers and facilitators can help to successfully implement TDM‐guided dosing of all oral oncolytics.

HCPs are an important stakeholder in the implementation of TDM‐guided dosing. Many of the identified barriers are the result of a lack of awareness and knowledge. HCPs in the survey agreed that they lack experience with TDM‐guided dosing, though they did recognize the added value of TDM‐guided dosing in selected cases, such as old fragile patients. However, they request further prospective evidence proving the clinical benefits of routine TDM‐guided dosing. In relation to this request, medical oncologists are reluctant to perform dose reductions in case of a high C_trough_ and limited side effects. However, it is questionable whether a prospective randomized controlled trial studying the efficacy of TDM‐guided dosing is feasible since TDM‐guided dosing is already part of routine practice in an increasing number of hospitals. HCPs are also not fully aware of the actual costs of analysis of (external) blood samples and of reimbursement of TDM‐guided dose adjustments within label. In conclusion, education of HCPs on TDM could be an important tool to overcome many of these beforementioned barriers. The advantage of educating HCPs in the implementation of a healthcare innovation has been described in previous literature, for example concerning the implementation of TDM‐guided dosing of vancomycin. The appropriateness of vancomycin dosing increased from 51% to 78% after an education‐based intervention.^33^, ^34^ However, little evidence is available regarding the optimal education strategies^34^, ^35^ and these may differ between HCP groups, such as pharmacists, physicians, or nurses.^36^ During the interviews, it was suggested that knowledge of TDM‐guided dosing could be increased by incorporating TDM into training programs of HCPs, especially of medical oncologists, since hospital pharmacists are well known with the concept of TDM‐guided dosing due to their experience with other types of drugs.^17^, ^18^ This latter statement could not be confirmed in the survey however, since 48.5% and 24.2% of hospital pharmacists and medical oncologists, respectively, considered themselves as having a lack of experience with TDM for TKIs. Therefore, TDM‐guided dosing should be incorporated into education of all HCPs.

Survey results showed many barriers in the domain of the patients. These barriers are related to a lack of knowledge and awareness of the possibilities of TDM‐guided dosing and fears, such as fear of a lack of efficacy in case of a low C_trough_. Lack of knowledge in patients and a negative attitude of patients are well known as barriers for implementing optimal healthcare in literature.^37^, ^38^ To overcome these barriers, survey results suggested patients can be informed properly about TDM‐guided dosing either by their treating physician or via patient advocacy groups. Therefore, patients should be informed about TDM primarily by their medical oncologist and patient advocacy groups should cooperate with medical oncologists to provide correct and nuanced information.

An important barrier in the domain of organizational context was the duration of processing time for the measurement and interpretation of the C_trough_ result. In most hospitals, no bioanalytical method to quantify TKI plasma concentrations is available. Therefore, it requires time to send the blood sample to another hospital, to analyze and interpret the result, to report the result back to the referring hospital and to interpret it there. This turnaround time from sample collection to reporting of the result could be reduced by providing an overview of when and where C_trough_ measurements are being performed, as suggested in the survey. Furthermore, as stated by patients, blood samples should be obtained well ahead of the appointment with the medical oncologist.

Finally, in the finances, law, and governance domain, survey respondents suggested to arrange separate financial compensation for the analyses of TKIs. However, HCPs estimated the analytical costs of TDM measurement to be higher than the actual low prices, especially in regard to total costs of treatment (e.g., costs of the TKI, scans, and hospital visits). It has been demonstrated for imatinib that TDM‐guided dosing is cost‐effective in treating patients with GIST.^39^ With the (upcoming) loss of patency for some TKIs, cost‐effectiveness will even be improved.^39^

This study has several strengths. First, we identified barriers and facilitators in all six domains of the theoretical framework of Grol and Wensing and interviewed all stakeholders involved. We included HCPs and patients until data saturation was reached and focused the interview guide on TKIs for which TDM‐guided dosing has shown to be of added value and feasible in clinical practice. Therefore, we consider our data to be complete and specific. By conducting a nationwide survey among both HCPs and patients, we were able to quantitatively determine the most important barriers and facilitators in order to determine an implementation strategy. Furthermore, by using this framework, we are able to translate our findings to all oral oncolytics with scientific support for TDM‐guided dosing, since they are not specific for the three drugs involved.

This study has some limitations as well. First, we were not able to calculate a survey response rate because of privacy regulations of the Dutch Society of Medical Oncology and the Dutch Association of Hospital Pharmacists. However, the coverage of hospitals that treat patients with solid tumors with imatinib, sunitinib, and pazopanib in the survey was 79% and especially hospitals that treat a large number of patients were represented in the survey. Second, due to the differences in health care systems and the application of TDM‐guided dosing in other countries, it might be difficult to extrapolate part of our results to other countries. On the contrary, themes such as awareness, knowledge, and education may be considered to play a role more universally.

In conclusion, we identified and quantified barriers and facilitators enabling a successful strategy to implement TDM‐guided dosing of oral oncolytics for patients with solid tumors. We would recommend to focus this strategy on educating both HCPs and patients and to reduce turnaround time from sample collection to reporting of results.

## AUTHOR CONTRIBUTIONS

**Kim Westerdijk:** Conceptualization (equal); data curation (equal); formal analysis (equal); methodology (equal); writing – original draft (equal); writing – review and editing (equal). **Neeltje Steeghs:** Conceptualization (equal); methodology (equal); writing – review and editing (equal). **Casper S. J. Tacke:** Data curation (equal); formal analysis (equal); writing – original draft (equal). **Winette T. A. van der Graaf:** Conceptualization (equal); methodology (equal); writing – review and editing (equal). **Nielka P. van Erp:** Conceptualization (equal); methodology (equal); writing – review and editing (equal). **Gerard van Oortmerssen:** Conceptualization (equal); writing – review and editing (equal). **Rosella P. M. G. Hermens:** Conceptualization (equal); data curation (equal); formal analysis (equal); methodology (equal); writing – original draft (equal); writing – review and editing (equal). **Ingrid M. E. Desar:** Conceptualization (equal); formal analysis (equal); methodology (equal); writing – original draft (equal); writing – review and editing (equal).

## FUNDING INFORMATION

This study was part of the TUNE project (grant no. 11575) funded by the Dutch Cancer Society (KWF Kankerbestrijding).

## CONFLICT OF INTEREST STATEMENT

N.S. provided consultation or attended advisory boards for Boehringer Ingelheim, Cogent Biosciences, Ellipses Pharma, Incyte, Luszana. N.S. received research grants from Abbvie, Actuate Therapeutics, Amgen, Array, Ascendis Pharma, AstraZeneca, Bayer, Blueprint Medicines, Boehringer Ingelheim, BridgeBio, Bristol‐Myers Squibb, Cantargia, CellCentric, Cogent Biosciences, Cresecendo Biologics, Cytovation, Deciphera, Dragonfly, Eli Lilly, Exelixis, Genentech, GlaxoSmithKline, IDRx, Immunocore, Incyte, InteRNA, Janssen, Kinnate Biopharma, Kling Biotherapeutics, Luszana, Merck, Merck Sharp & Dohme, Merus, Molecular Partners, Navire Pharma, Novartis, Numab Therapeutics, Pfizer, Relay Pharmaceuticals, Revolution Medicine, Roche, Sanofi, Seattle Genetics, Taiho, Takeda. All outside the submitted work, all payment to the Netherlands Cancer Institute. W.G. has received institutional research fees from Lilly and advisory compensation from Springworks, PTC Therapeutics and Agenus all to the institute.

N.E. received grants for the conduct of investigator driven studies from Ipsen and Astellas.

The other authors declare no conflict of interest.

## ETHICS STATEMENT

The current study was approved by the local ethical committee of the Radboudumc in Nijmegen, the Netherlands (dossier number 2019–5631 and 2022‐16004).

## CONSENT

Informed consent was obtained from all subjects involved in the study.

## CLINICAL TRIAL REGISTRATION NUMBER

Not applicable.

## Supporting information


Figure S1.

Table S1.

## Data Availability

The data presented in this study are available on request from the corresponding author. The data are not publicly available due to privacy restrictions.
