# Supplementary material for: Therapeutic drug monitoring to personalize dosing of imatinib, sunitinib, and pazopanib: A mixed methods study on barriers and facilitators
Source: Cancer Med. 2023 Oct 30;12(22):21041–56. doi: 10.1002/cam4.6663 (PMC10709747; doi:10.1002/cam4.6663)
Supplement: Supplementary file 1 — Figure S1. [file CAM4-12-21041-s001.pdf]

Barriers	Facilitators
**Domain: innovation itself**<br>"At that time we missed an easy and practical overview of when to take blood samples, how to interpret them, when to repeat them and what to do in case of an inadequate exposure."	".. but clinically you will only see the results of the treatment after at least three months and with TDM, you can evaluate the exposure in an earlier stage and adjust the dose if the patient is underdosed. "
**Domain: healthcare professional**<br>"I don't think there is enough literature supporting applying routine TDM in clinical practice for all patients."<br>"I don't think that everyone is convinced of the added value."<br>"So, they know about the drugs and adverse events, but they don't know that well about the possibilities of TDM and that TDM actually optimizes treatment."	"I think you should focus on persuading the medical oncologist in particular."<br>"I think you should make sure that the awareness of TDM is present in the people who should arrange this. Then, I think most HCPs would be willing to use TDM, because why wouldn't they?"
**Domain: patient**<br>"If my dose would be increased due to TDM, the first thing that would cross my mind would be… what if I start getting side-effects?"<br>"When you come and see the doctor and the TDM result is not there… That is an important problem. You already go to the appointment with a lot of stress and when you leave the room, you still don't know anything."	"If I see that value goes up after my dose has been increased, then I have a bit more reassurance that the drug will still do its work."<br>"Maybe the doctor can send me an email to remind me to skip my TKI. Or I can remember myself: I put a reminder in my phone or a write a note and put it on the table."<br>"I think if you explain to patients that they have to skip their TKI and why this is important, that this will be doable in clinical practice.."
**Domain: social context**<br>"I was inclined to do TDM guided dosing… But in the hospital where I work now, my colleagues were not and the laboratory did not know what to do and where to send the blood sample to. I can imagine that someone would easily skip TDM in this situation."	"Yes, our pharmacy and the hospital pharmacists are very involved. If we bring up a case with our thoughts, then they are willing to think together with us to come up with an answer."
**Domain: organizational context**<br>"Well, I think that the availability of the relevant clinical information is a crucial bottleneck. The HCP that establishes the dose advise needs this information to properly provide a dose advice."<br>"If the TDM results takes too long, then it is a bit too little too late."	"Maybe the analysis for the C_{trough} result can be performed more often, so that you can have a result available within one week."<br>"..I know the day of the analysis of everything that I outsource. So I can adjust my planning to this."
**Domain: finance, law and governance**<br>"That is the difference between academic and smaller hospitals. We have less resources available and have to be more efficient."<br>"I think the costs of TDM are a limiting factor. That physicians are unaware of the financial compensation for the TDM measurement and therefore decide not to do it."	: "Once you see the use and added value, it's a different story, isn't it? Then the worries about costs are less."<br>"It would be nice if we arrange financial compensation for a few TDM measurements for each patient."
