# Supplementary material for: Therapeutic drug monitoring to personalize dosing of imatinib, sunitinib, and pazopanib: A mixed methods study on barriers and facilitators
Source: Cancer Med. 2023 Oct 30;12(22):21041–56. doi: 10.1002/cam4.6663 (PMC10709747; doi:10.1002/cam4.6663)
Supplement: Supplementary file 2 — Table S1. [file CAM4-12-21041-s002.docx]

**Supporting Information**

**Supplemental Table 1 – Overview of health care professionals included in the interviews**

| **Hospital** | **Region** | **Number of medical oncologists** | **Number of hospital pharmacists** |
| --- | --- | --- | --- |
| *Academic* | North | 1 | 1 |
| *Teaching* | North | 1 | 0 |
| *Teaching (n=2)* | East | 2 | 2 |
| *Academic* | South | 1 | 1 |
| *Teaching* | South | 1 | 1 |
| *Non-teaching* | South | 1 | 1 |
| *Teaching* | West | 0 | 1 |
| *Non-teaching (n=2)* | West | 2 (1 nurse practitioner) | 2 |

**Supplemental Table 2. Responses from HCP questionnaire**

| **Barrier / facilitator** | **Medical oncologist**† | **Hospital pharmacist**† | **Questions / theses** | Agree (%) | Neutral (%) | Disagree (%) |
| --- | --- | --- | --- | --- | --- | --- |
| Barrier | X | X | I think there is a lack of scientific evidence for performing TDM-guided dosing. | 24.2 | 7.6 | 68.2 |
| Barrier | X | X | I do not see added value of performing TDM-guided dosing on a routine basis. | 19.7 | 12.1 | 68.2 |
| Barrier | X | X | Prospective studies proving the added value of TDM-guided dosing on treatment outcome are required before I will perform TDM-guided dosing myself. | 40.9 | 22.7 | 36.4 |
| Barrier | X | X | As long as there is no evidence that TDM is cost-effective, I will not perform TDM-guided dosing. | 9.1 | 25.8 | 65.2 |
| Barrier | X | X | A disadvantage of TDM is that patients have to take a variable number of tablets. | 15.2 | 27.3 | 57.6 |
| Barrier | X | X | TDM can lead to frequent dose adjustments, which can be confusing for patients. | 43.9 | 16.7 | 39.4 |
| Barrier | X | X | I am not aware of the possibility to perform TDM-guided dosing for TKIs. | 9.1 | 1.5 | 89.4 |
| Barrier | X | X | I lack knowledge on TDM-guided dosing with TKIs. | 25.8 | 18.2 | 56.1 |
| Barrier | X | X | I do not know where I can find background information on TDM-guided dosing. | 15.2 | 21.2 | 63.6 |
| Barrier |  | X | I am not aware of the availability of TDM monographs for TKIs. | 12.1 | 3 | 84.8 |
| Barrier | X | X | I think I lack experience with TDM with TKIs. | 36.4 | 10.6 | 53 |
| Barrier |  | X | I think it is difficult to translate a C_trough_ level to a dose advice for an individual patient. | 27.3 | 12.1 | 60.6 |
| Barrier | X |  | It is difficult to translate a dose advice to clinical practice when the C_trough_ does not correlate with clinical presentation and the dose advice does not seem fitting. | 42.4 | 21.2 | 36.4 |
| Barrier | X |  | I think patients are not aware of the possibility of performing TDM-guided dosing. | 78.8 | 15.2 | 6.1 |
| Barrier | X |  | I think TDM-guided dosing is difficult to understand for patients. | 21.2 | 12.1 | 66.7 |
| Barrier | X |  | I think patients question the added value of TDM. | 6.1 | 24.2 | 69.7 |
| Barrier | X |  | I think patients experience stress prior to the C_trough_ result, especially when the result is not available at the appointment with the medical oncologist. | 30.3 | 27.3 | 42.4 |
| Barrier | X |  | I think patients fear an increase in adverse events and a decrease in quality of life in case of dose escalation. | 27.3 | 21.2 | 51.5 |
| Barrier | X |  | I think patients are afraid of a lack of efficacy (for instance in case of low C_trough_ or in case of a dose reduction based on TDM). | 63.6 | 6.1 | 30.3 |
| Barrier | X |  | I think family members of patients fear dose adjustments (both increase in adverse events due to dose escalation or lack of efficacy in case of dose reduction). | 27.3 | 33.3 | 39.4 |
| Barrier | X |  | I do not think patients are aware of the possibility of performing TDM blood sampling closer to home. | 60.6 | 27.3 | 12.1 |
| Barrier | X |  | I think creating awareness of TDM-guided dosing through patient advocacy groups can cause anxiety among patients. | 30.3 | 33.3 | 36.4 |
| Barrier | X | X | I would not perform TDM-guided dosing since it has not been implemented in clinical practice guidelines / local protocols. | 18.2 | 16.7 | 65.2 |
| Barrier | X | X | It is difficult to retrieve information on how to practically perform TDM-guided dosing (such as timing of blood sampling, what tubes, what to do with dose adjustments). | 21.2 | 16.7 | 62.1 |
| Barrier | X | X | I am reluctant to perform TDM-guided dosing since I am stuck in a routine and TDM has not been incorporated in that routine yet. | 25.8 | 12.1 | 62.1 |
| Barrier | X | X | I consider the workload to be too high to perform TDM-guided dosing. | 7.6 | 9.1 | 83.3 |
| Barrier | X | X | I am reluctant to perform TDM-guided dosing since this is not routine practice in colleagues. | 21.2 | 9.1 | 69.7 |
| Barrier | X | X | I think it is difficult to request a blood sample due to the absence of the proper order for the C_trough_ measurement. | 18.2 | 10.6 | 71.2 |
| Barrier | X | X | I think it is difficult to request a blood sample due to the absence of one universal national laboratory form. | 18.2 | 6.1 | 75.8 |
| Barrier | X | X | There was no lab order available when the patient was in the hospital for blood sampling. | 10.6 | 27.3 | 62.1 |
| Barrier | X | X | Laboratory workers do not know what to do with blood sample and where to send it to. | 22.7 | 22.7 | 54.5 |
| Barrier | X | X | I am not aware of the possibility of collecting blood samples closer to a patients' home. | 27.3 | 16.7 | 56.1 |
| Barrier | X |  | I am not aware that patients can not take their TKI right before blood sampling and that I should instruct them accordingly. | 9.1 | 15.2 | 75.8 |
| Barrier | X | X | Patients forget to adjust their TKI intake prior to blood sample, despite the fact that they were instructed to do so. | 43.9 | 27.3 | 28.8 |
| Barrier | X | X | The result of the C_trough_ measurement takes too long. | 40.9 | 22.7 | 36.4 |
| Barrier |  | X | I think it is difficult to formulate a dose advice since information regarding last intake or clinical information (such as toxicity) is lacking. | 27.3 | 21.2 | 51.5 |
| Barrier | X | X | There is no clear contact person in case I have questions. | 16.7 | 9.1 | 74.2 |
| Barrier | X | X | I think there is a lack of expertise with TDM-guided dosing in my hospital pharmacy. | 9.1 | 15.2 | 75.8 |
| Barrier | X | X | I think it is difficult that TDM requires collaboration between hospital pharmacists and medical oncologists. | 18.2 | 12.1 | 69.7 |
| Barrier | X |  | I feel resistance to consult an external hospital pharmacist when my own hospital does not have a hospital pharmacist with an expertise in TDM-guided dosing. | 3 | 24.2 | 72.7 |
| Barrier | X | X | I am reluctant with decreasing the dose of a TKI based on TDM in case of a high C_trough_ and limited side effects. | 43.9 | 21.2 | 34.8 |
| Barrier | X | X | I think the TDM blood samples are too expensive. | 12.1 | 37.9 | 50 |
| Barrier | X | X | I am concerned the expenses of my department will increase due to TDM. | 19.7 | 19.7 | 60.6 |
| Barrier | X | X | I am concerned that the expenses of the hospital will increase due to blood samples, while reduction in costs due to dose reductions will only benefit health insurance companies. | 31.8 | 31.8 | 36.4 |
| Barrier | X | X | I did not know that dose adjustments based on TDM are paid for by the health insurance companies. | 45.5 | 13.6 | 40.9 |
| Barrier | X | X | I believe that external blood samples are more expensive compared to blood samples analyzed at the local hospital. | 53 | 7.6 | 39.4 |
| Barrier |  | X | It is a problem that the costs for TDM-guided dosing are especially at expense of the pharmacy. | 39.4 | 15.2 | 45.5 |
| Facilitator | X | X | I feel confident that an adequate C_trough_ represents an adequate treatment. | 62.1 | 25.8 | 12.1 |
| Facilitator | X | X | The benefit of TDM-guided dosing is that you can optimise treatment with a TKI (by increasing efficacy and decreasing toxicity). | 89.4 | 4.5 | 6.1 |
| Facilitator | X | X | The benefit of TDM is that you can optimise treatment at an early stage. | 83.3 | 10.6 | 6.1 |
| Facilitator | X | X | The benefit of TDM is that you can individualise treatment. | 87.9 | 10.6 | 1.5 |
| Facilitator | X | X | The benefit of TDM is that you can start at a low dose and increase the dose carefully based on C_trough_ level in case of fragile patients. | 68.2 | 21.2 | 10.6 |
| Facilitator | X | X | The benefit of TDM is that you can objectify whether a dose of a TKI results in adequate exposure. | 84.8 | 7.6 | 7.6 |
| Facilitator | X | X | The benefit of TDM is that you can evaluate therapy adherence. | 60.6 | 27.3 | 12.1 |
| Facilitator | X | X | The benefit of TDM-guided dosing is that you can measure the effect of interacting medication on the exposure of the TKI. | 95.5 | 3 | 1.5 |
| Facilitator | X |  | The benefit of TDM is that the hospital pharmacist evaluates possible medication interactions when interpreting a C_trough_ result. | 84.8 | 9.1 | 6.1 |
| Facilitator | X |  | I think TDM increases the experience of receiving maximum treatment effort. | 75.8 | 15.2 | 9.1 |
| Facilitator | X |  | I think TDM increases patient satisfaction. | 51.5 | 42.4 | 6.1 |
| Facilitator | X |  | I think TDM increases patient involvement. | 60.6 | 18.2 | 21.2 |
| Facilitator | X |  | It would facilitate TDM if patients ask their treating physician for TDM-guided dosing. | 48.5 | 24.2 | 27.3 |
| Facilitator | X |  | I think patient advocacy groups can increase awareness of patients for TDM. | 54.5 | 24.2 | 21.2 |
| Facilitator | X |  | It would facilitate TDM if a patients' partner or family asks for TDM-guided dosing. | 36.4 | 33.3 | 30.3 |
| Facilitator | X | X | I think TDM is easy to perform in daily practice. | 72.7 | 16.7 | 10.6 |
| Facilitator |  | X | In case of an external Ctrough measurement, the results should be communicated to the hospital pharmacist of the referring hospital instead of the medical oncologist. | 90.9 | 9.1 | 0 |
| Facilitator | X | X | It would facilitate TDM if timing of TKI intake and blood samples would be standardized, for example by instructing patients to take their medication in the evening, so they do not have to skip their TKI intake prior to blood sampling. | 63.6 | 28.8 | 7.6 |
| Facilitator | X | X | It would facilitate TDM if timing of TKI intake and blood samples would be standardized, for example by taking blood samples exactly 12 or 24 hours after TKI intake. | 33.3 | 33.3 | 33.3 |
| Facilitator | X | X | It would facilitate TDM if surrounding hsopitals are performing TDM-guided dosing. | 63.6 | 12.1 | 24.2 |
| Facilitator | X | X | Concerns of HCPs about costs of TDM can be overcome by convincing HCPs of the added value of TDM guided dosing. | 87.9 | 7.6 | 4.5 |
| Facilitator | X | X | It would facilitate TDM if pharmacology of TKIs is implemented in education of health care professionals. | 95.5 | 4.5 | 0 |
| Facilitator | X | X | It would facilitate TDM to inform both HCPs and patients adequately about the processing time of the TDM result. | 90.9 | 6.1 | 3 |
| Facilitator | X | X | It would facilitate TDM if an overview would be available of where C_trough_ measurements are being performed. | 86.4 | 3 | 10.6 |
| Facilitator | X | X | It would facilitate TDM if one dedicated nurse would collect all the blood samples. | 21.2 | 19.7 | 59.1 |
| Facilitator | X | X | It would facilitate TDM if patients would be performed about TDM-guided dosing by the pharmacy when they are handed over the TKI for the first time. | 69.7 | 15.2 | 15.2 |
| Facilitator | X | X | The implementation of TDM-guided dosing can be enhanced by arranging separate financial compensation for costs of TDM. | 86.4 | 9.1 | 4.5 |
| Facilitator | X | X | The implementation of TDM-guided dosing can be enhanced when health insurance companies incorporate TDM-guided dosing as a quality requirement. | 83.3 | 6.1 | 10.6 |
| Facilitator | X | X | The implementation of TDM-guided dosing can be enhanced when TDM-guided dosing is incorporated in the drug label by the pharmaceutical company. | 83.3 | 7.6 | 9.1 |
| Facilitator |  | X | Modeling programs would be an advantage to be able to predict the effect of a dose adjustment to the exposure of the TKI. | 81.8 | 9.1 | 9.1 |
| Facilitator | X | X | I think Dried Blood Splot analysis would improve accessibility and use of TDM-guided dosing. | 74.2 | 15.2 | 10.6 |

Abbreviations: C_trough_, trough concentration; HCPs, healthcare professionals; TDM, Therapeutic Drug Monitoring; TKI, Tyrosine Kinase Inhibitor;

† an ‘X’ marks whether the question was to be answered by medical oncologists and/or by hospital pharmacists.

**Supplemental Table 3. Responses from patient questionnaire**

| Barrier / facilitator | Questions / theses | Agree (%) | Neutral (%) | Disagree (%) |
| --- | --- | --- | --- | --- |
| Barrier | I think it is difficult that TDM can lead to variations in the dose of imatinib. | 34.5 | 27.6 | 37.9 |
| Barrier | Before this questionnaire I was not aware of the possibility of TDM. | 39.7 | 8.6 | 50.0 |
| Barrier | I experience stress prior to the TDM result. | 29.3 | 15.5 | 53.4 |
| Barrier | I am afraid of having more side effects when the dose of imatinib is increased based on TDM. | 70.7 | 6.9 | 20.7 |
| Barrier | I am afraid for a decrease in quality of life when the dose of imatinib is increased based on TDM. | 72.4 | 10.3 | 17.2 |
| Barrier | I am afraid of having less effect of imatinib when the dose is decreased based on TDM. | 37.9 | 13.8 | 48.3 |
| Barrier | When the imatinib concentration in my blood is low, I am afraid of having less effect of imatinib. | 56.9 | 13.8 | 29.3 |
| Barrier | My partner or family is concerned when my imatinib dose is adjusted based on the imatinib concentration in my blood. | 27.6 | 37.9 | 29.3 |
| Barrier | The practical aspects of TDM, such as blood sampling and adjusting the moment of imatinib intake to blood sampling, causes inconvenience to me. | 24.1 | 22.4 | 53.4 |
| Barrier | I think TDM is driven by financial stimuli. | 8.6 | 15.5 | 74.1 |
| Barrier | I am under the impression that my doctor is not aware of the possibility of TDM. | 1.7 | 20.7 | 75.9 |
| Barrier | I think my doctor lacks knowledge on TDM. | 1.7 | 20.7 | 74.1 |
| Barrier | I think my doctor lacks experience with TDM. | 1.7 | 24.1 | 72.4 |
| Barrier | I am under the impression that my doctor thinks TDM is expensive. | 1.7 | 27.6 | 69.0 |
| Barrier | I am under the impression that my doctor thinks TDM is difficult. | 1.7 | 22.4 | 74.1 |
| Barrier | I am not aware of the possibility of performing TDM blood sampling close to home. | 50.0 | 13.8 | 34.5 |
| Barrier | I was not instructed properly by the doctor or nurse to adjust my imatinib intake before blood sampling. | 25.9 | 29.3 | 39.7 |
| Barrier | I sometimes forget to adjust my imatinib intake before blood sampling. | 5.2 | 37.9 | 50.0 |
| Barrier | There was no lab order available when I was in the hopsital for blood sampling. | 13.8 | 32.8 | 46.6 |
| Barrier | I was not asked about my last imatinib intake when I was in the hospital for blood sampling. | 22.4 | 25.9 | 46.6 |
| Barrier | It takes too long before the TDM result is available. | 29.3 | 31.0 | 34.5 |
| Barrier | I think the adjustments in my imatinib dose based on TDM are confusing. | 8.6 | 46.6 | 37.9 |
| Facilitator | I think it would help the implementation if the doctor discusses expectations of patients of TDM (for example how long it takes before the TDM result is available. | 91.4 | 1.7 | 6.9 |
| Facilitator | I think it would help the implementation if I would receive a clear and simple explanation on TDM. | 87.9 | 3.4 | 8.6 |
| Facilitator | I think it would help the implementation if the result of the imatinib concentration is available when I have my apppointment with my doctor. | 84.5 | 5.2 | 10.3 |
| Facilitator | I think I understand enough what TDM is. | 82.8 | 12.1 | 5.2 |
| Facilitator | I think it would help the implementation if patients would receive information on TDM through patient organizations. | 82.8 | 6.9 | 10.3 |
| Facilitator | I think TDM is an adequate way to determine whether I am receiving the right dose of imatinib. | 77.6 | 10.3 | 12.1 |
| Facilitator | I expect my doctor to be openminded about using TDM. | 75.9 | 6.9 | 15.5 |
| Facilitator | I see added value of TDM. | 74.1 | 10.3 | 15.5 |
| Facilitator | I think it would help the implementation if there is a clear contact person in case I have questions about TDM. | 70.7 | 6.9 | 20.7 |
| Facilitator | I think the effect of imatinib increases with TDM. | 69.0 | 8.6 | 22.4 |
| Facilitator | I think I am using the correct dose of imatinib with TDM. | 69.0 | 15.5 | 12.1 |
| Facilitator | I think I am receiving the best treatment with TDM. | 67.2 | 15.5 | 15.5 |
| Facilitator | I am more involved in my treatment with TDM. | 63.8 | 10.3 | 24.1 |
| Facilitator | I think it would help the implementation if I would be reminded to adjust my imatinib intake before blood sampling. | 62.1 | 22.4 | 13.8 |
| Facilitator | I think the side effects of imatinib treatment decrease with TDM. | 60.3 | 13.8 | 25.9 |
| Facilitator | I think it would help the implementation if patients themselves ask their doctors for TDM. | 60.3 | 19.0 | 19.0 |
| Facilitator | I am more satisfied with my treatment with TDM. | 56.9 | 12.1 | 29.3 |
| Facilitator | I think I will experience less side effects with TDM. | 44.8 | 27.6 | 25.9 |
| Facilitator | My partner or family sees added value of TDM. | 41.4 | 13.8 | 44.8 |
| Facilitator | I think my adherence to taking my imatinib every day increases with TDM. | 13.8 | 70.7 | 13.8 |
| Final conclusion | I would like my doctor to use TDM during my imatinib treatment. | 62.0 | 27.6 | 10.3 |

**Figure legends**

**Supplemental Figure 1. Illustrative quotations from participants during interviews**
